# Introduction to the Special Section on Healthcare for Space, Space for Healthcare

**DOI:** 10.1109/OJEMB.2023.3309591

**Published:** 2023-08-29

**Authors:** Ilaria Cinelli

**Affiliations:** Aerospace Medical Association 267031 Alexandria VA 22314-3579 USA

## Abstract

The International Space Station (ISS) has been critical in increasing our understanding of space exploration and healthcare during the last two decades. As the only long-lasting microgravity laboratory in space, the ISS has provided a unique environment for researchers to conduct experiments that would be impossible on Earth. These discoveries have aided astronauts in staying healthy in space and have resulted in new treatments and therapies for humans on Earth. With more services dependent on space assets and the public's inclusion in commercial human spaceflight, new prospects in healthcare are emerging, and space is becoming the next frontier in our quest for improved health.

The International Space Station (ISS) has been critical in increasing our understanding of space exploration and healthcare during the last two decades. As the only long-lasting microgravity laboratory in space, the ISS has provided a unique environment for researchers to conduct experiments that would be impossible on Earth. These discoveries have aided astronauts in staying healthy in space and have resulted in new treatments and therapies for humans on Earth. With more services dependent on space assets and the public's inclusion in commercial human spaceflight, new prospects in healthcare are emerging, and space is becoming the next frontier in our quest for improved health.

The Special Section of the IEEE Open Journal of Engineering in Medicine and Biology, entitled “Healthcare for Space, Space for Healthcare,” is devoted to exploring healthcare's broad and interdisciplinary nature and its connection to space. It aims to raise awareness of the numerous educational, research, and technical opportunities that emerge from investigating the reciprocal impact of space on healthcare and vice versa. “*Healthcare for Space*” encompasses health-related initiatives to study human health in space or on Earth. These initiatives can be conducted in space, transferred to space, or designed specifically for space. Space medicine and space biology are two scientific areas that match the “Healthcare for Space” theme. “*Space for Healthcare*” refers to health-related activities that utilise space assets, advancements in space technology, or simulations of specific characteristics of the space environment to study human health on Earth or in space. Research areas such as disaster management, public health, and ground space research are encompassed within this theme. This Special Section highlights recent efforts and encourages innovation in this dynamic field. By documenting the latest advancements and promoting collaboration between different research fields, it strives to enhance our understanding of human health in space and on Earth.

Medical innovation in spaceflight is critical for guaranteeing astronaut safety and well-being and promoting healthcare innovation on Earth. The challenges posed by space operations directly impact the development of medical technology and practices, making space exploration a significant catalyst for medical innovation [A1]. This impact will be further amplified by upcoming government-led human exploration programs on the Moon, as the retirement of the ISS and the establishment of new space infrastructure drive innovation to ensure the long-term sustainability of human activities beyond Low Earth Orbit (LEO) [A1]. During spaceflight, it is imperative to establish and maintain healthcare capabilities throughout the mission. Presently, space medicine operations rely on terrestrial support for managing medical events aboard the ISS. However, as we venture beyond LEO in future missions, the crew themselves will increasingly assume responsibility for onboard medical care. Consequently, there is a growing need to develop advanced medical capabilities that empower crew members to independently manage their health without relying solely on support from Earth [A2].

Digital technology holds great promise for providing advanced care during space missions. For example, tele-pharmacy services, facilitated by digital platforms for teleassistance and pharmaceutical teleconsulting, are expanding and have intriguing applications for spaceflight [A3]. Other technologies, such as exergames and virtual reality, have the potential to promote psychological and psychosocial health by immersing astronauts in stimulating surroundings and scenarios [A4]. Furthermore, biomedical hardware originally developed for terrestrial applications has the potential to be utilised in space environments. For example, Ishikita invented a minimally invasive sputum aspiration device during the coronavirus public health emergency and has been approved as a Class I medical device in Japan [A5]. While such a device could help advance astronaut care during future operations, regulatory protocols may still be necessary to ensure its compatibility with spaceflight. Seyedmadani et al. emphasised the importance of rigorous regulatory processes to prove the compatibility of biomedical instrumentation with spaceflight [A6]. It is crucial to align new space preventive measures, created independently of governmental techniques, with existing regulations and best practices [A6].

Ground-based technologies and facilities are employed to test novel technologies and gain new insights into physiological effects under fractional Earth gravity. Berengueres et al. hypothesised that adding sound transparency to a space suit prototype improves cognitive performance and that augmenting and processing environmental sounds may lead to establishing novel countermeasures for surface exploration activities [A7]. Then, Song et al. investigated human brain activity in an aqueous environment that replicated Moon gravity to explore the cortical area involved in maintaining balance in low gravity [A8]. Nevertheless, access to ground facilities for conducting such experiments presents challenges that necessitate updates in terms of accessibility and cost reduction. These efforts are crucial to encourage broader participation in research and development, supporting forthcoming human activities beyond LEO. To boost the impact of terrestrial space research and access to space biology and astrobiology, Chu et al. created the EuniceScope, a low-cost reconfigurable 3D-printed microscope [A9]. This device democratises astrobiology, mainly for educational purposes, highlighting the significant need to modernise ground-based microgravity-simulating platforms.

In alignment with the aforementioned requirements for improvement, there is a pressing need to advance the legal framework that bridges the domains of healthcare and space. Specifically, attention should be given to the provision of space medicine on the ground. Zhou et al. focused on Canada and emphasised the significance of updating legal healthcare support frameworks to match the rising demand for local physicians to provide space healthcare [A10]. As space exploration and research continue to evolve, it is essential to ensure that healthcare innovation and regulation keep pace with these developments.

Overall, the intersection of medical innovation and space exploration has significant implications for healthcare on Earth, as well as for advancing our understanding of human physiology and developing new technologies to improve healthcare delivery in space. The potential benefits of space-based healthcare innovations are significant and continually evolving. As technology advances, new opportunities that could transform healthcare on Earth and in space will arise.


Ilaria Cinelli, Guest Editor
Aerospace Medical Association
Alexandria, VA 22314 USA
i_cinelli@yahoo.it


## Appendix Related Articles

[A1] I. Cinelli, “How space operations drive innovation in human healthcare,” *IEEE Open J. Eng. Med. Biol.*, vol. 4, pp. 158–161, 2023, doi: 10.1109/OJEMB.2023.3244042.
[A2] D. R. Levin et al., “Enabling human space exploration missions through progressively earth independent medical operations (EIMO),” *IEEE Open J. Eng. Med. Biol.*, vol. 4, pp. 162–167, 2023, doi: 10.1109/OJEMB.2023.3255513.
[A3] M. A. dos Santos, J. Herbert, I. Cinelli, J. A. L. Burmann, V. V. Soares, and T. Russomano, “Development of a digital platform: A perspective to advance space telepharmacy,” *IEEE Open J. Eng. Med. Biol.*, vol. 4, pp. 168–172, 2023, doi: 10.1109/OJEMB.2023.3237988.
[A4] G. Ciocca and H. Tschan, “The enjoyability of physical exercise: Exergames and virtual reality as new ways to boost psychological and psychosocial health in astronauts. A prospective and perspective view,” *IEEE Open J. Eng. Med. Biol.*, vol. 4, pp. 173–179, 2023, doi: 10.1109/OJEMB.2023.3234014.
[A5] N. Ishikita, “Novel 3D printable aspiration device SPUTA VACUUMER designed to minimise invasion,” *IEEE Open J. Eng. Med. Biol.*, vol. 4, pp. 180–183, 2023, doi: 10.1109/OJEMB.2023.3248875.
[A6] K. Seyedmadani, T. A. Keith, R. Baraquiel, A. M. Yasemin, A. Metin, and F. A. Jennifer, “Processes for designing innovative biomedical hardware to use in space and on earth,” *IEEE Open J. Eng. Med. Biol.*, vol. 4, pp. 184–189, 2023, doi: 10.1109/OJEMB.2023.3270393.
[A7] J. Berengueres, M. AlKuwaiti, M. Abduljabbar, and F. Taher, “Adding sound transparency to a spacesuit: Effect on adding sound transparency to a spacesuit: Effect on cognitive performance in females,” *IEEE Open J. Eng. Med. Biol.*, vol. 4, pp. 190–194, 2023, doi: 10.1109/OJEMB.2023.3288740.
[A8] S. Song and A. D. Nordin, “Balance perturbations in simulated low-gravity modulate human premotor and frontoparietal electrocortical theta, alpha, and beta band spectral power,” *IEEE Open J. Eng. Med. Biol.*, vol. 4, pp. 195–202, 2023, doi: 10.1109/OJEMB.2023.3238319.
[A9] W. Y. Chu and K. K. Tsia, “EuniceScope: Low-cost imaging platform for studying microgravity cell biology,” *IEEE Open J. Eng. Med. Biol.*, vol. 4, pp. 203–210, 2023, doi: 10.1109/OJEMB.2023.3257991.
[A10] A. Zhou, V. Nwaokoro, V. Oosterveld, and A. Sirek, “Building a one country one licensure framework: Applications for the future of Canadian space physicians,” *IEEE Open J. Eng. Med. Biol.*, vol. 4, pp. 211–214, 2023, doi: 10.1109/OJEMB.2023.3241597.

